# Free l-glutamate-induced modulation in oxidative and neurochemical profile contributes to enhancement in locomotor and memory performance in male rats

**DOI:** 10.1038/s41598-020-68041-y

**Published:** 2020-07-08

**Authors:** Saiqa Tabassum, Saara Ahmad, Syeda Madiha, Sidrah Shahzad, Zehra Batool, Sadia Sadir, Saida Haider

**Affiliations:** 1 Neurochemistry and Biochemical Neuropharmacology Research Unit, Department of Biochemistry, University of Karachi, Karachi, 75270 Pakistan; 2 Department of Biosciences, Faculty of Life Science, Shaheed Zulfiqar Ali Bhutto Institute of Science and Technology (Szabist), Karachi, Pakistan; 3 Department of Biological and Biomedical Sciences, Aga Khan University Hospital, Karachi, Pakistan; 4 Dr. Panjwani Center for Molecular Medicine and Drug Research, International Center for Chemical and Biological Sciences, University of Karachi, Karachi, 75270 Pakistan

**Keywords:** Cognitive neuroscience, Learning and memory, Biochemistry, Neuroscience

## Abstract

Glutamate (Glu), the key excitatory neurotransmitter in the central nervous system, is considered essential for brain functioning and has a vital role in learning and memory formation. Earlier it was considered as a harmful agent but later found to be useful for many body functions. However, studies regarding the effects of free l-Glu administration on CNS function are limited. Therefore, current experiment is aimed to monitor the neurobiological effects of free l-Glu in male rats. l-Glu was orally administered to rats for 5-weeks and changes in behavioral performance were monitored. Thereafter, brain and hippocampus were collected for oxidative and neurochemical analysis. Results showed that chronic supplementation of free l-Glu enhanced locomotor performance and cognitive function of animals which may be attributed to the improved antioxidant status and cholinergic, monoaminergic and glutamatergic neurotransmission in brain and hippocampus. Current results showed that chronic supplementation of l-Glu affects the animal behaviour and brain functioning via improving the neurochemical and redox system of brain. Free l-Glu could be a useful therapeutic agent to combat neurological disturbances however this requires further targeted studies.

## Introduction

Central nervous system (CNS) modulates behaviour and brain functioning with the help of multiple neurotransmitters. One of the important neurotransmitters is Glutamate (Glu)^1^ which is a nonessential amino acid (AA) present abundantly in the body^2^. It is involved in a variety of body functions either directly or by converting into other products^3^. Evidence shows the importance of Glu in facilitating protein synthesis^4^, intestinal nutrition, cell signalling, gene expression modulation, immune responses^5^, regulating blood glucose level, removing excess ammonia^6^, disposal of excess or waste nitrogen^7^ as well as in intermediary metabolism^2^ as an important energy fuel^5^. Other than this, it is also a precursor of various biologically active components like glutathione (GSH), GABA, purine and pyrimidine nucleotides, polyamines, poly-glutamated folate cofactors or certain AAs like glutamine, alanine, aspartate, proline, arginine, citrulline^2–5,8^. Glu is present in various foods either as naturally occurring free Glu (vegetable, seafood, milk, meat, cheese) or as a by-product of hydrolysed protein (used in seasoning) or as a salt; MSG (monosodium glutamate), a food additive or flavouring agent in canned foods, dry mixes, sauces, and soups^9^.

Nutritionally, Glu is important for taste perception and for facilitation of gastric and pancreatic function^3,9^. Various studies have reported the potential health effects of dietary glutamate supplementation that include improvement of taste and palatability^10^, modification of gastric secretion and motility, enhanced cellular proliferation^11^, stimulation of gastro-intestinal exocrine secretions and prevention of incidence of diarrhoea^12^. It is also used to treat dyspepsia^13^, improves growth performance, enhances protein synthesis^4,14^, restores mucous circulation and AA metabolism, and prevents cellular injury and apoptosis of enterocytes^4^. Reports have also shown its potential in amelioration of endotoxin-induced intestinal damage and maintaining intestinal integrity^5,14^, increasing nucleotide synthesis^3^, improving antioxidant status^15^, ROS scavenging^16^, amelioration of hypoxia-induced oxidative stress^17^, inhibition of fat accumulation^18^ and progression of atherogenesis and fatty liver disease^19^.

Glu metabolism is shown to be mainly involved in maintaining normal brain function^20^ to regulate neurogenesis, synaptogenesis, neurite outgrowth and neuronal survival^21^. Most of the brain Glu is synthesised either from glucose or 3-hydroxybutyrate or from other AAs^8^. High concentration of Glu is present in blood and CSF^22^ and in brain regions responsible for mediating memory and cognitive functioning like cortex, hippocampus and striatum^1,23^. Synaptic Glu signalling is involved in neuronal growth and synaptic plasticity^24^ contributing to learning and memory processing and cognitive functioning^1,2,25,26^. Its usefulness in facilitation of potassium transport across blood brain barrier (BBB) suggests its promising role in future treatment of neurological conditions^6^. Glu neurons are widely distributed in forebrain and hippocampus^1^. Hippocampus is reported to be largely dependent upon Glu signalling implicated in learning and memory functions^24^. Supplements of Glu are available in market for various purposes such as to maintain blood glucose levels, to increase immunity, to build proteins, and to promote optimal absorption and assimilation^27–29^. It was initially thought that its systemic or oral administration cannot affect its availability in CNS as Glu cannot cross the BBB^28,30^. But later researchers found that Glu can cross the BBB up to a certain extent and dietary Glu supplements improve the brain functioning^31,32^ leading to generation of hypothesis that chronic free Glu supplementation at a dose equivalent to average daily intake of humans might have beneficial effect on behaviour of animals. Despite extensive beneficial use in periphery, studies addressing its beneficial effects on CNS are limited. Since to date studies on the use of free l-Glu on CNS function are scarce, hence the objective of the present study is therefore specifically to investigate the effects of free Glu on brain and associated neurobehavioral alterations at a dose equivalent to adequate human intake in rats. In order to understand the underlying mechanism responsible for behavioural alterations, the current study was further aimed to monitor the effects of dietary free Glu supplements on motor and cognitive performance as well as associated changes in brain redox status. Glu/GABA content and monoaminergic and cholinergic neurotransmission in rats was also studied.

## Results

**Figure 1:**
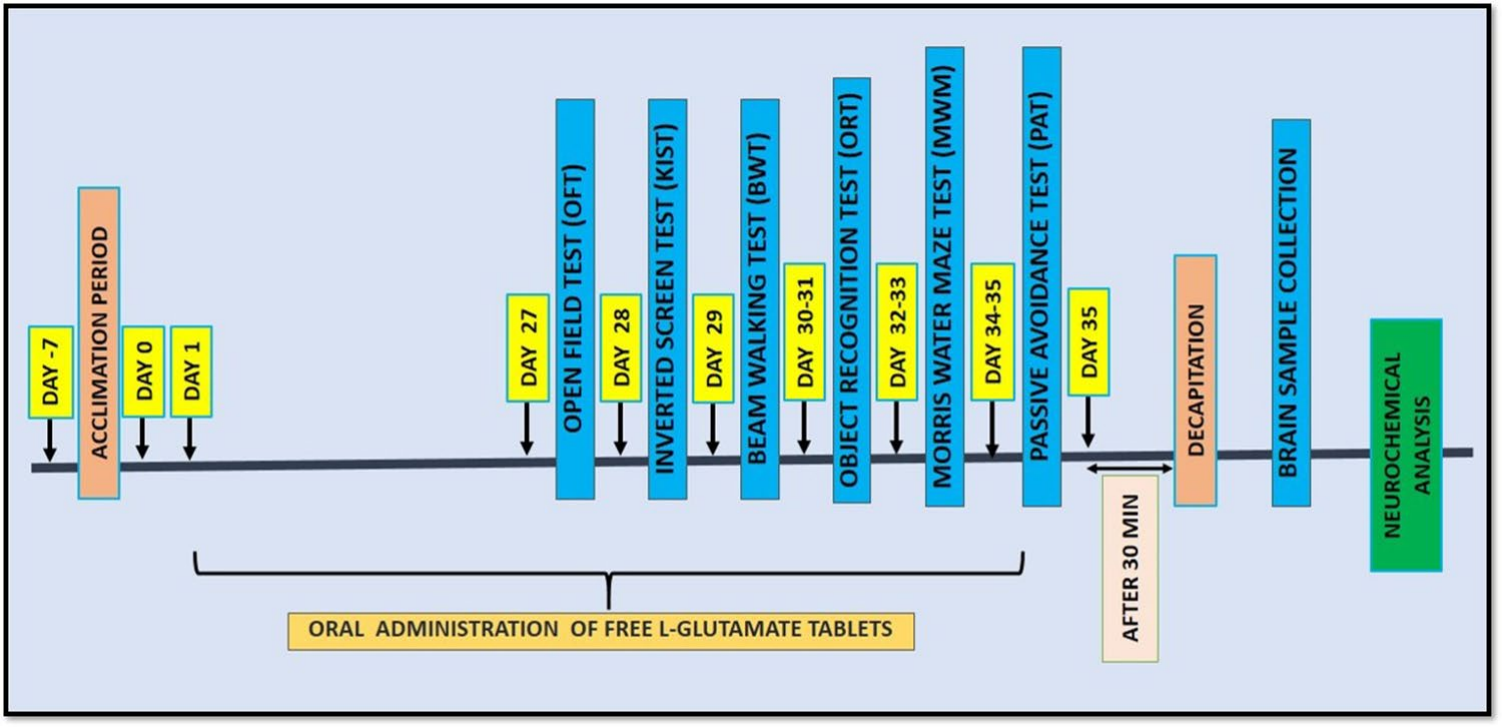
Schematic representation of treatment schedule and experimental design.

The aim of the current study is to find out the effects of free l-Glu on locomotor activity and learning and cognitive functioning along with monitoring alterations in oxidative profile and neurochemical content in the brain as shown in Fig. 1. Along with behavioural the body weight and food intake of rats was also monitored throughout the experiment. The effects of Glu supplementation on the body weight and food intake were not different compared to control (Table 1).

**Table 1:** Effects of dietary free glutamate supplementation on weekly food intake and the body weight of animals during treatment. Data presented as mean ± SD (n = 6).

	Food intake		Body weight	
	Control	Glutamate	Control	Glutamate
Initial	12.65 ± 1.93	11.85 ± 0.82	160.17 ± 10.03	160.11 ± 11.11
Week 1	11.46 ± 0.72	10.84 ± 1.21	163.67 ± 10.98	161.33 ± 11.52
Week 2	10.92 ± 0.67	11.55 ± 1.95	165.05 ± 12.55	163.17 ± 11.28
Week 3	11.41 ± 1.55	11.91 ± 1.68	166.43 ± 12.62	165.83 ± 12.73
Week 4	11.82 ± 1.12	11.90 ± 1.38	168.33 ± 13.03	167.67 ± 13.92
Week 5	10.72 ± 0.75	10.67 ± 0.27	168.67 ± 12.69	169.66 ± 13.62

A non-significant effect was obtained by the repeated measure analysis when compared with control rats.

### Effect of free l-Glu on locomotor performance

**Figure 2:**
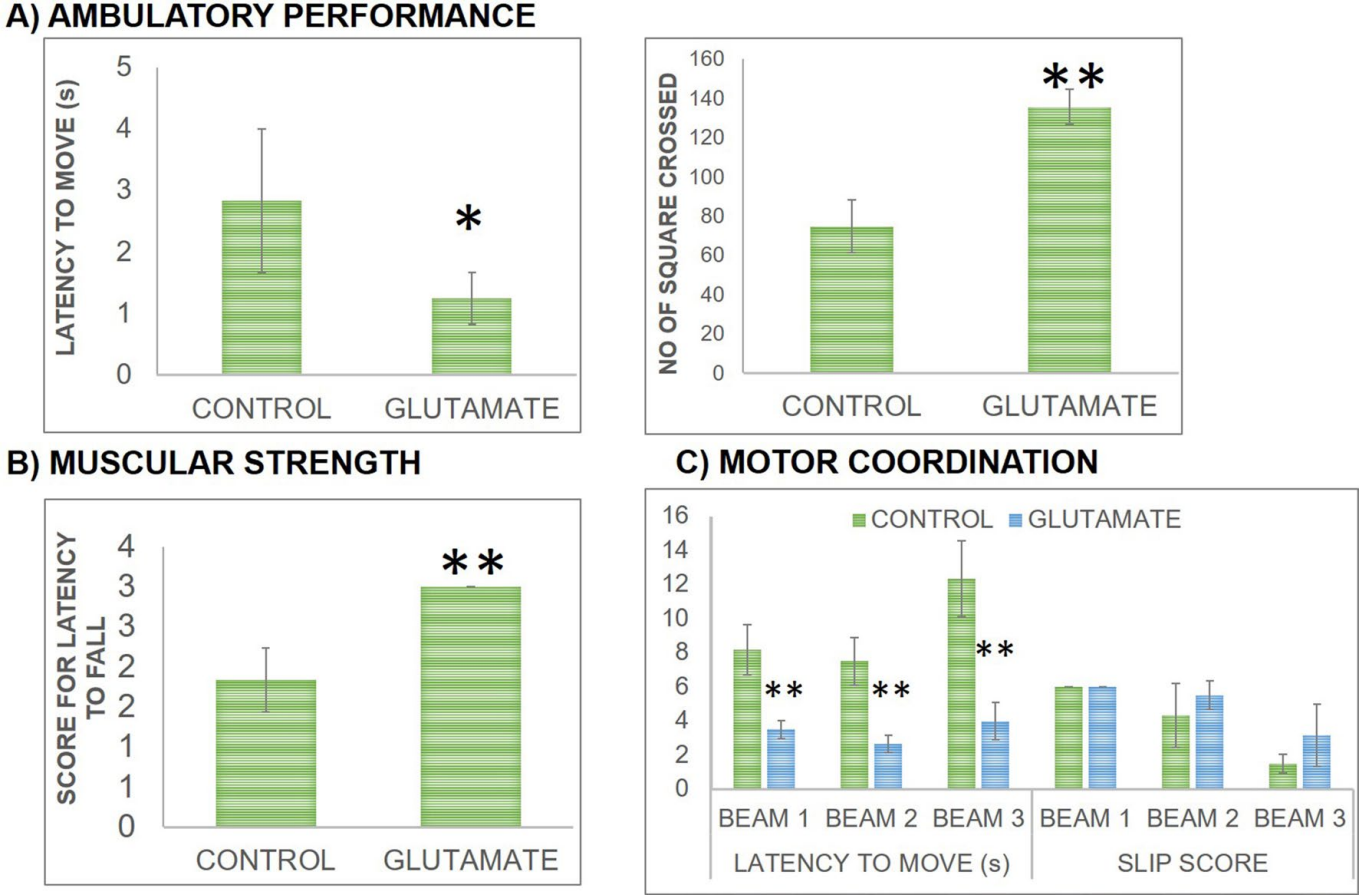
Effect on locomotor activity was evaluated via monitoring **(a)** ambulatory performance which is evaluated by open field test in terms of latency (s) to move from the central square (left) the number of squares crossed (right), **(b)** muscular strength evaluated by Kondziela’s Inverted screen test in terms of latency (s) to fall from the inverted screen and **(c)** motor coordination evaluated by Beam walking test in terms of latency (s) to cross the beam and number of foot slips off the beam on three different beam sizes (3 cm, 2 cm and 1 cm). For each group n = 6 and values are presented as mean ± S.D. All significant differences are expressed as * *P* < 0.05, ** *P* < 0.01 compared to control group.

Effect of Glu supplementation on rat’s ambulatory performance was assessed in OFT in terms of latency to move from the central square and the number of squares crossings made during 5 min time span which is shown in Fig. 2. Observation showed that ambulatory performance was improved following Glu supplementation compared to controls as evident by significant decline in latency to move (Fig. 2a) (*t *(10) = 2.875, *P* < 0.05) and significant increase in square crossings (*t *(10) = 11.41, *P* < 0.01) during 5 min time span (Fig. 2a) compared to controls.

Effect of Glu supplementation on muscular strength of rats was evaluated in KIST by recording the latency to fall from the inverted screen during 2 min time span which is presented in Fig. 2b. It was observed that muscular strength of rats was increased following Glu supplementation compared to controls as a significant increment in latency time to fall from inverted screen was observed. Statistical analysis showed a significant (U = *P* < 0.01) difference between groups with a mean rank of 9.5 for Glu group compared to controls having mean rank of 3.5.

Effect of Glu supplementation on motor coordination and balance in rats was assessed by beam walking test by recording latency time taken to cross the beam and number of foot slips off the beam on three different beam sizes (3 cm, 2 cm and 1 cm) during 2 min time span which is presented in Fig. 2c. It was observed that motor coordination and balance in rats was increased following Glu treatment compared to controls as evident by significant reduction in latency time to cross the beam and an increase in score for foot slips off the beam. There is a significant decline in latency time for beam of 3 cm width (*t *(10) = 7.278, *P* < 0.01), for beam of 2 cm width (*t *(10) = 8.043, *P* < 0.01) and for beam of 1 cm width (*t *(10) = 8.154, *P* < 0.01) as shown in Fig. 2c. Data analysis of score for foot slips off the beam revealed that there was no significant difference in score for foot slips off the beam in Glu supplemented group over beam of 3 cm, 2 cm and 1 cm width comparable to control group.

### Effect of free l-Glu on learning and memory function

**Figure 3:**
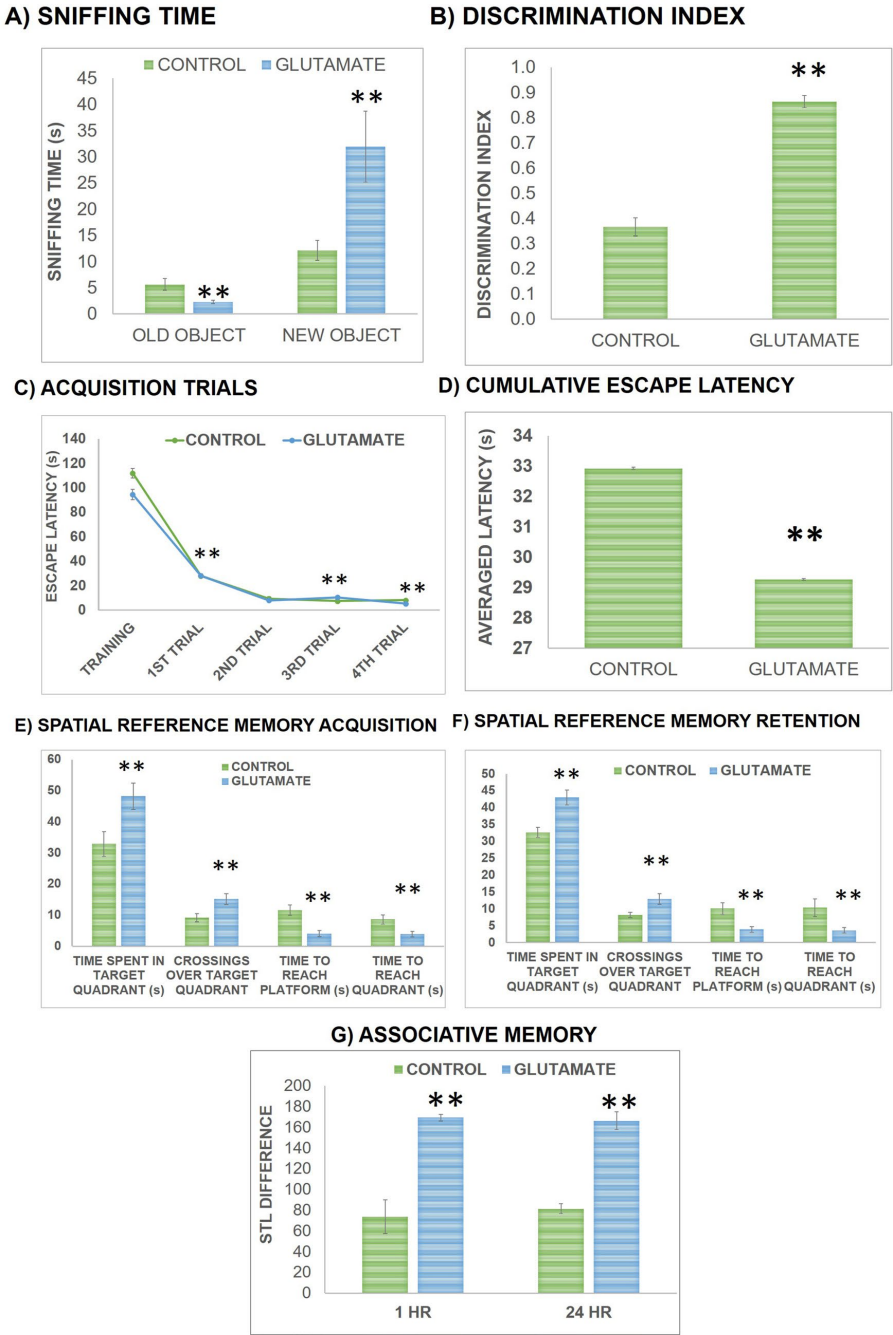
Effect on memory performance is presented; (1) Recognition memory in terms of **(a)** sniffing time for familiar (old) and novel object and **(b)** discrimination index; (2) Spatial memory performance in terms of **(c)** escape latencies of acquisition training trials, **(d)** averaged escape latencies, **(e)** spatial reference memory acquisition (1 h probe trial) and **(f)** retention (24 h probe trial) by monitoring escape latency time, latency to find the target quadrant (NW), duration of time spent (seconds) in the target quadrant and the number of entries made by rat over target quadrant; (3) Associative memory performance in terms of **(g)** step-trough latency difference. For each group n = 6 and values are presented as mean ± S.D. Significant differences were expressed as ** *P* < 0.01 compared to control group and for training trials ** *P* < 0.01 compared to control and ++ *P* < 0.01 compared to trial 1.

Recognition memory was monitored by ORT by noting the sniffing time for novel and familiar objects followed by computing discrimination index that is shown in Fig. 3a and b. Observations showed that recognition memory was improved following Glu supplementation as a significant enhancement was seen in sniffing time for new object and in discrimination index. Statistical analysis showed significant decline (*t *(10) = 7.022, *P* < 0.01) in sniffing time for old object following Glu supplementation in comparison to control group while sniffing time for novel object was significantly increased (*t *(10) = 6.896, *P* < 0.01) in Glu group in comparison to control group as shown in Fig. 3a. Discrimination index data showed a significant enhancement in (*t (10)* = 28.01, *P* < 0.01) Glu group in comparison to controls as shown in Fig. 3b.

The differences in escape latencies during 4 acquisition trials showed significant effect of trials (F (4, 40) = 287.536, *P* < 0.01), group (F (1, 10) = 42.04, *P* < 0.01) and interaction between trials x groups (F (4, 80) = 9.918, *P* < 0.01). Pair-wise comparisons showed a significant decline over trials in escape latencies of animals which decreased significantly (*P* < 0.01) (displayed in Fig. 3c) indicating that memory performance was enhanced in animals over trials. For cumulative escape latency of all acquisition trials a significant increase was observed in Glu group (t = 6.484, *P* < 0.01) in comparisons to controls as shown in Fig. 3d. Data analysis of reference memory parameters during 1 h probe trial showed significant decline in platform latency (escape latency time to reach platform location) (*t *(10) = 9.449, *P* < 0.01) and in target quadrant latency (*t *(10) = 6.919, *P* < 0.01) while significant enhancement in duration of time spent in target quadrant (*t *(10) = 6.471, *P* < 0.01) and in number of entries made over target quadrant (*t *(10) = 6.755, *P* < 0.01) as shown in Fig. 3e. Statistical analysis of reference memory parameters during 24 hr probe trial also revealed significant decline in platform latency (*t *(10) = 7.656, *P* < 0.01) and in target quadrant latency (*t *(10) = 5.82, *P* < 0.01), and a significant enhancement in duration of time spent in target quadrant (*t *(10) = 9.399, *P* < 0.01) and in number of entries in target quadrant (*t *(10) = 6.874, *P* < 0.01) in comparison to controls as presented in Fig. 3f.

**Figure 4:**
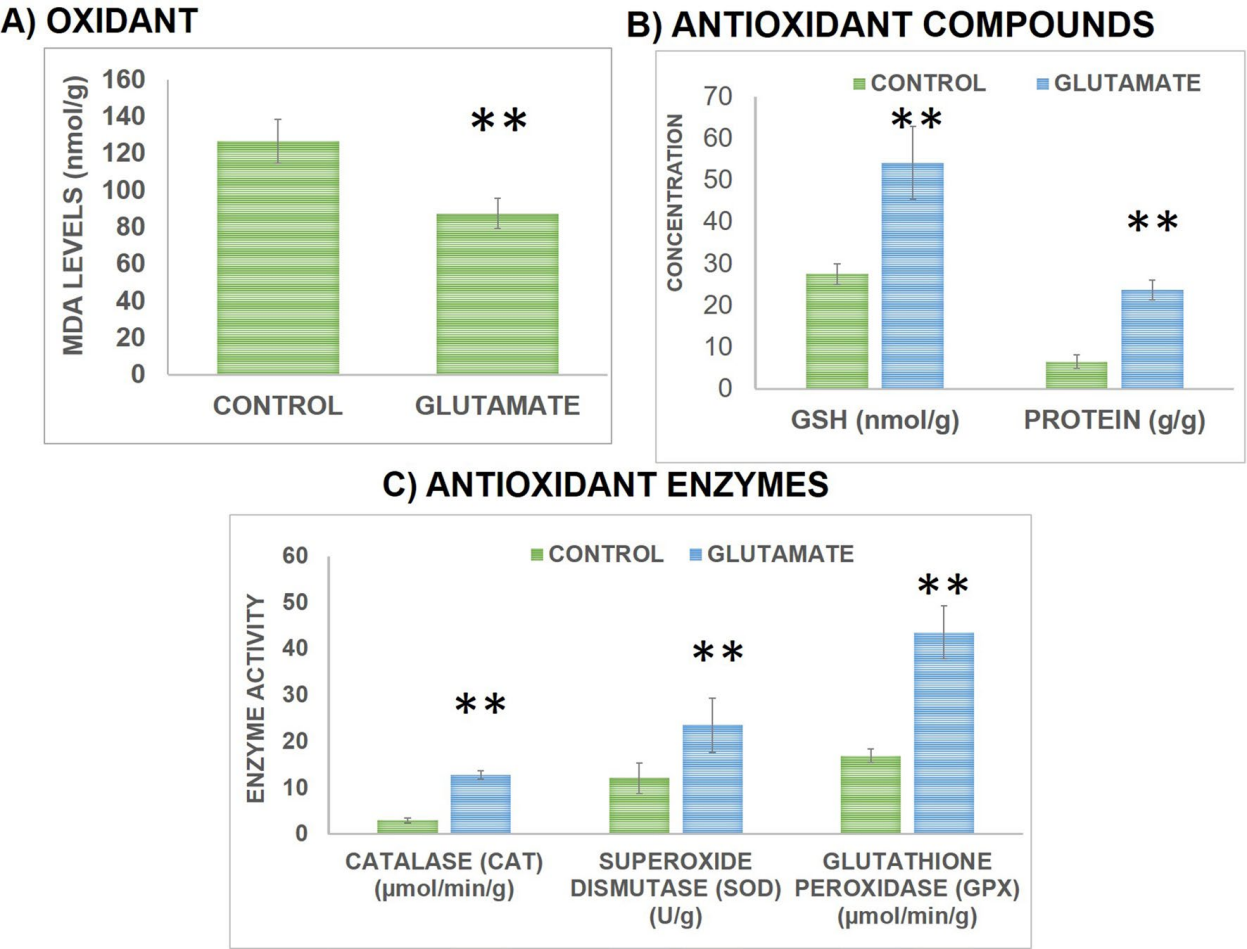
Effect on oxidative profile of rats was assessed via determining the levels of major oxidant (MDA) **(a)**, levels of antioxidant (GSH and Protein) compounds **(b)** and levels of antioxidant enzymes (CAT, GPx, SOD) **(c)**. For each group n = 6 and values are presented as mean ± S.D. Significant differences were expressed as ** *P* < 0.01 compared to control group.

Associative memory performance was evaluated using PAT by recording step-through latency to enter into the dark chamber during both training and test phases by evaluating the difference between pre- and post-training step-through latencies presented in Fig. 3g. Observations showed a significant improvement in associative memory following Glu supplementation as evident by increase in difference between step-through latencies. Statistical analysis showed a significant increment in difference between step-through latencies of Glu group during 1 h. (*t *(10) = 14.106, *P* < 0.01) and 24 h. (*t *(10) = 21.1, *P* < 0.01) sessions in comparison to control group as presented in Fig. 4. On the basis of results, it can be suggested that following the intake of Glu tablets associative memory was improved.

### Effect of chronic administration of free l-Glu on oxidative status of brain

Effects of Glu supplementation on oxidative status of brain was assessed via estimating the lipid peroxidation (MDA) content, antioxidant enzyme activities (CAT, GPx and SOD) and content of antioxidant compounds (GSH and protein) in brain which is presented in Fig. 4. The MDA levels were significantly (*t *(10) = 6.679, *P* < 0.01) reduced in Glu group in comparison to controls as presented in Fig. 4a. Data analysis on content of antioxidant compounds (GSH and Protein) showed a significant increment in GSH levels (*t *(10) = 7.144, *P* < 0.01) and protein content (*t *(10) = 14.437, *P* < 0.01) in Glu group in comparison to controls as presented in Fig. 4b. Moreover, data analysis of activities of antioxidant enzymes (CAT, GPx and SOD) showed a significant enhancement in activities of CAT (*t *(10) = 21.769, *P* < 0.01), GPx (*t *(10) = 11.101, *P* < 0.01) and SOD (*t *(10) = 4.174, *P* < 0.01) in Glu group compared to controls as presented in Fig. 4c. From the findings it can be suggested that following Glu treatment oxidative status of brain was improved.

### Effect of chronic administration of free l-Glu on neurochemical profile of brain

**Figure 5:**
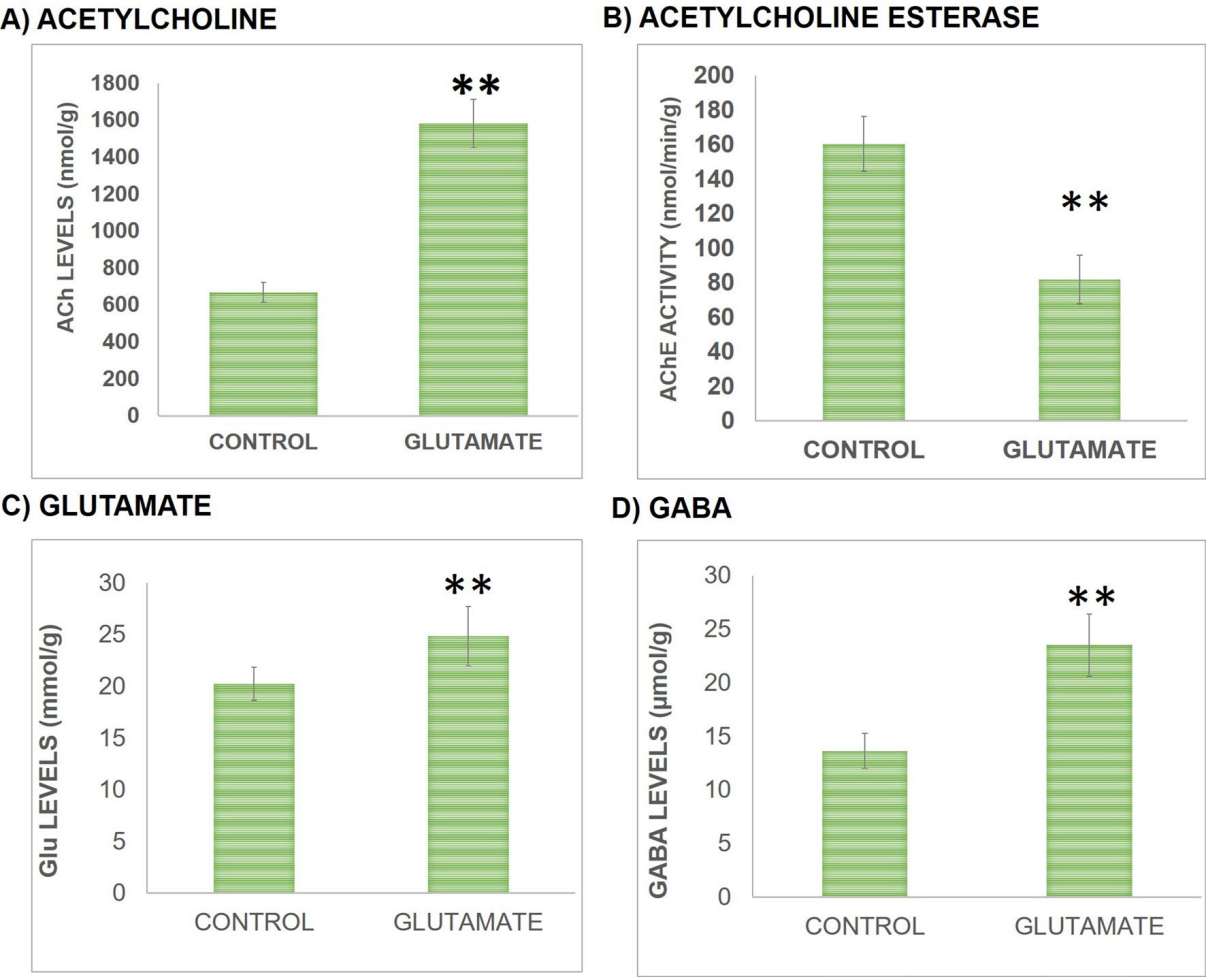
Effect on cholinergic and glutamatergic profile of rats was assessed via determining the levels of ACh content **(a)** and AChE activity** (b)** and the levels of hippocampal Glu **(c)** and GABA **(d)**. For each group n = 6 and values are presented as mean ± S.D. Significant differences were expressed as ** *P* < 0.01 compared to control group.

Cholinergic status of brain was monitored via estimating ACh content and AChE activity. Results showed enhancement in ACh content following Glu supplementation. Significant increment (*t *(10) = 7.73, *P* < 0.01) in ACh content was observed in Glu group in comparison to controls as presented in Fig. 5a. While in AchE activity a significant reduction (*t *(10) = 14.437, *P* < 0.01) was seen following Glu supplementation compared to controls as shown in Fig. 5b.

Effect of Glu supplementation on Glu and GABA levels in hippocampus was also determined. It was observed that Glu and GABA hippocampal content was altered following Glu treatment as there is a significant increment in Glu (*t *(10) = 4.36, *P* < 0.01) and GABA (*t *(10) = 6.45, *P* < 0.01) levels in hippocampus of Glu group compared to controls as shown in Fig. 5c, d.

**Figure 6:**
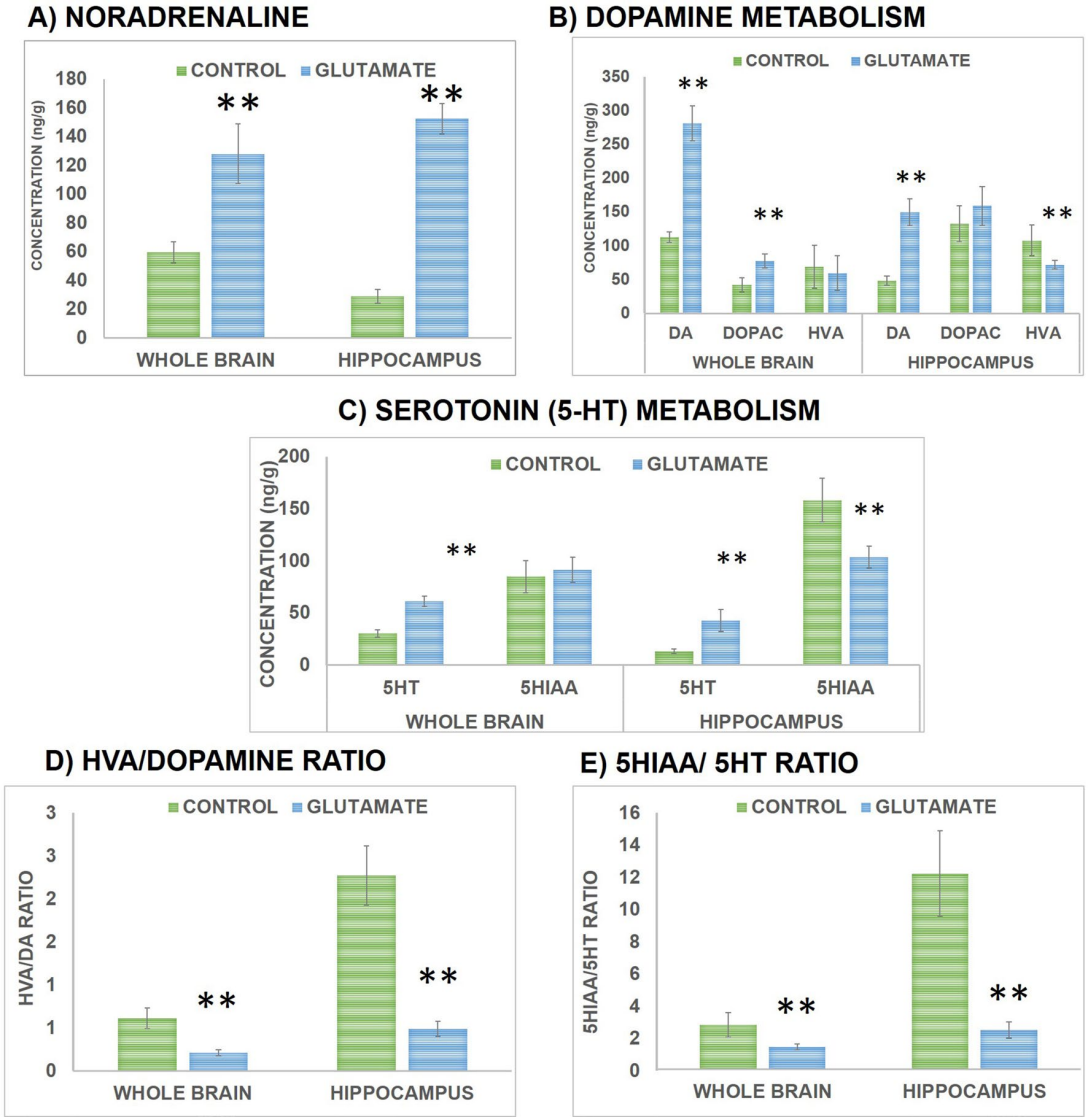
Effect on brain and hippocampal monoaminergic profile of rats was assessed via determining the levels of monoamines [NA **(a)**, DA **(b)**, 5-HT **(c)**] and its metabolites [DOPAC **(b)**, HVA **(b)**, 5-HIAA **(c)**] and ratio HVA/DA ratio **(d)** and 5HIAA/5HT **(e)**. For each group n = 6 and values are presented as mean ± S.D. Significant differences were expressed as ** *P* < 0.01 compared to control group.

Effect of Glu supplementation on monoaminergic profile of brain and hippocampus was determined via estimating the levels of NA, DA, 5-HT and its metabolites (DOPAC, HVA and 5-HIAA) using high pressure liquid chromatography coupled to electrochemical detection (HPLC-EC) method. It was observed that monoaminergic profile was altered following Glu treatment. There is a significant increment in NA concentration in brain (*t *(10) = 4.84, *P* < 0.01) and hippocampus (*t *(10) = 25.823, *P* < 0.01) of Glu group compared to controls as shown in Fig. 6a. Regarding DA metabolism there is a significant rise in concentration of DA (*t *(10) = 3.47, *P* < 0.01) and DOPAC (*t *(10) = 5.96, *P* < 0.01) in brain following Glu treatment compared to controls while HVA levels remained comparable. However, in hippocampus significant increase in DA levels (*t *(10) = 11.87, *P* < 0.01) and significant decline in HVA levels (*t *(10) = 3.72, *P* < 0.01) was observed following Glu treatment in comparison to controls whereas no significant change was observed in DOPAC levels as presented in Fig. 6b. 5-HT content was significantly increased in both brain (*t *(10) = 12.46, *P* < 0.01) and hippocampus (*t *(10) = 6.62, *P* < 0.01) following Glu treatment compared to controls while levels of 5-HIAA were significantly (*t *(10) = 5.73, *P* < 0.01) decreased in hippocampus after Glu intake whereas brain 5-HIAA levels remained unaltered as shown in Fig. 6c. Furthermore, the ratios of HVA/DA and 5HIAA/5HT were also computed in brain and hippocampus and a significant decline was observed in ratio of HVA/DA in both brain (*t *(10) = 7.87, *P* < 0.01) and hippocampus (*t *(10) = 12.24, *P* < 0.01) and in the ratio of 5HIAA/5HT in both brain (*t *(10) = 7.87, *P* < 0.01) and hippocampus (*t *(10) = 12.24, *P* < 0.01) indicating that monoamine neurotransmission was enhanced following Glu administration.

## Discussion

The current study found improvement in motor activity and learning and memory performance following chronic supplementation of free l-Glu via modulating the neurochemical and redox status in the brain. Reports showed that intake of l-Glu in drinking water had no effect on food intake and body weight^37,38,41^. Present findings support these studies as we also found no significant change in body weight and food intake of rats which might be associated with high energy expenditure. However present findings contradict with Yin et al.^15^ who reported increase in growth rate and body weight of pigs following dietary supplementation of 2% Glu for 7 days^15^. Glu being a major excitatory neurotransmitter is essential for all behaviors^21^. Our results showed that locomotor activity was improved following free Glu supplementation. Administration of Glu as a nootropic agent is a topic of interest since last 60 years. It is evident that Glu has a significant role in cognitive functioning and Glu signalling in brain contributes in synaptic maintenance and plasticity facilitating cognitive function^2,25^ and learning^1,26^. Reports showed that free L-Glu in brain is required for neuronal differentiation, migration and survival in developing brain by facilitating calcium transport contributing to memory enhancement via use-dependent alterations in synaptic efficacy that is involved in the formation and function of cytoskeleton^42^. But studies regarding the use of Glu as an adjunctive memory-enhancing agent^33^ lacks supporting experimental evidences. Present findings showed that chronic supplementation of free Glu at adequate amount enhances memory performance leading to improved spatial, recognition and associative memory processes. These findings are in agreement with the recent study which observed that umami taste in GI is responsible for stimulation of cortical and sub-cortical brain areas that are linked to working memory^40^. Moreover, studies showed that the effect of Glu depends upon its dosage. At low Glu levels intensity of Glu receptor is not high enough to cause excitotoxicity but indeed sufficient for memory enhancement^33^.

Oxidative stress, a state representing enhancement in levels of intracellular reactive oxygen species (ROS) that either act as free radicals themselves or breakdown to form free radicals^43,44^. Oxidative stress generation is attributed to disrupted balance between ROS generation and antioxidant scavenging activity^45,46^. ROS, the products of normal cellular metabolism attack PUFAs causing peroxidation of lipids (LPO) and generate MDA which is an important and sensitive marker of peroxidative damage^47^. This damage is overcome by the action of SOD that converts reactive superoxide anions to hydrogen peroxide which then by the action of CAT and GPx converts into water and molecular oxygen^48^. Along with these redox enzymes, certain endogenous antioxidants primarily GSH and proteins also provide defence against ROS via maintaining redox homoeostasis^48^. Studies have reported that ingested glutamate is transported to enterocytes via specific Na-dependent transporters and major amount (75–80%) is metabolized in the intestine by transamination, some amount (5–10%) enters into blood circulation, while 10–15% is converted to Glutamine and other bioactive molecules of sensory and signaling pathways^49,50^. Along with this free glutamate is also reported to have an important role in protein stability as it provides a negative charge^50^. Previously it has been observed that Glu addition to culture medium leads to enhanced cellular proliferation and membrane integrity and protects against oxidative stress^5^. A recent report also validated that Glu has ROS scavenging ability, so it is protective against oxidative stress^16^. Present findings also support this notion as we found reduced levels of MDA, the by-product of LPO in the brain of Glu-supplemented rats showing that it is protective against oxidative stress. Along with reduction in LPO, the GSH and total protein levels as well as antioxidant enzymes (SOD, CAT and GPx) activities also increased following chronic Glu supplementation. Previous researchers reported that the relationship between Glu and ROS is very complex^35,46^ as some have reported Glu to be beneficial in ameliorating oxidative stress while others reported it as a neurotoxic agent. However, current findings are in agreement with the reports that stated beneficial impact of l-Glu supplementation in ameliorating hypoxia-induced oxidative stress via reducing MDA levels and enhancing GSH levels in rats^17^, while in pigs it occurs via enhancing SOD levels and GSH content and inhibiting lipid peroxidation and MDA generation^15^. However, other reports showed that l-Glu supplementation either had no effect on SOD and CAT levels^1^ or failed to alleviate H_2_O_2_-induced oxidative stress^46^ producing oxidative damage^35^. It had little effect on increasing SOD and GPx concentration and decreasing MDA content in boars^46,51^. The antioxidant function of Glu as observed in current study might be due to the fact that it is the major substrate for GSH synthesis compared to cysteine and glycine^15,52,53^. GSH homoeostasis is considered to be essential for cellular defence against oxidative stress^15,52^ as it regulates redox state of cell and is involved in detoxification process in all cell types^5,54^. It can be depicted from previous reports that reduction in oxidative stress along with improved neurotransmission might be responsible for enhancement in cognitive retention capacity of animals^43^. Hence, the improvement in memory and retention capacity observed in present study might be attributed to the improved oxidative status of the brain following Glu administration in rats.

Cholinergic neurons play a key role in memory and attention^32,55^ and the neurotransmitter present in these neurons is acetylcholine (ACh). Evidence showed that both Glu and ACh play important role in memory^56^ as interactions between these neurotransmitters may be important for memory formation^57^. In particular, ACh might be involved in facilitating Glu activity via coordination of acquisition and recall states in cortex and hippocampus^57^. Intra-hippocampal infusion of receptor agonist of ACh and Glu also reported to improve retention while infusion of antagonists impaired retention^58^. The action of ACh is terminated upon its hydrolysis by the enzyme AChE thus reducing the availability of ACh in synapse and producing memory deficits^59^. In current study we found that supplementation of free Glu reduces the activity of AChE and enhances the ACh content in brain which might be responsible for improved cognitive performance. Further, we also determined the effect of Glu supplementation on monoaminergic neurotransmission in brain and found that Glu supplementation enhances concentration of NA, DA and 5-HT via affecting their metabolism in brain. It has been seen that HVA/DA (Fig. 6d) and 5HIAA/5HT ratio (Fig. 6e) is reduced following chronic Glu supplementation showing that turnover of DA and 5HT is reduced and their greater amount is available in synapse for performing their action which might be responsible for improved locomotor and cognitive function. Previous reports from our laboratory have shown that increased levels of monoamines (noradrenaline, dopamine and serotonin) paralleled the improvement in cognition and memory function^32,60^. Moreover, both Glu and GABA levels were increased following chronic Glu supplementation that may also be responsible for memory improvement in the present study. These findings contradict the previous studies reporting that Glu behaved as a neurotoxic agent impairing cognitive performance^34^, increasing oxidative stress^35^, damaging neuronal cells and producing excitotoxic lesions^36^ ultimately leading to neurodegenerative disorders^34,36^. However, later on researchers found free l-Glu as a beneficial agent^3,10–12,18,37–39^. Although Glu is found abundantly in foods^18^, but a brief review of all the reports regarding beneficial effect of free l-Glu shows that these beneficial effects are only addressed in periphery while effects on CNS are limited. Moreover, a recent report showed that umami taste (of Glu) activates para-hippocampal gyrus, an important memory retrieval area, that involves the modulation of working memory processing and contributes to efficient learning^40^.

**Figure 7:**
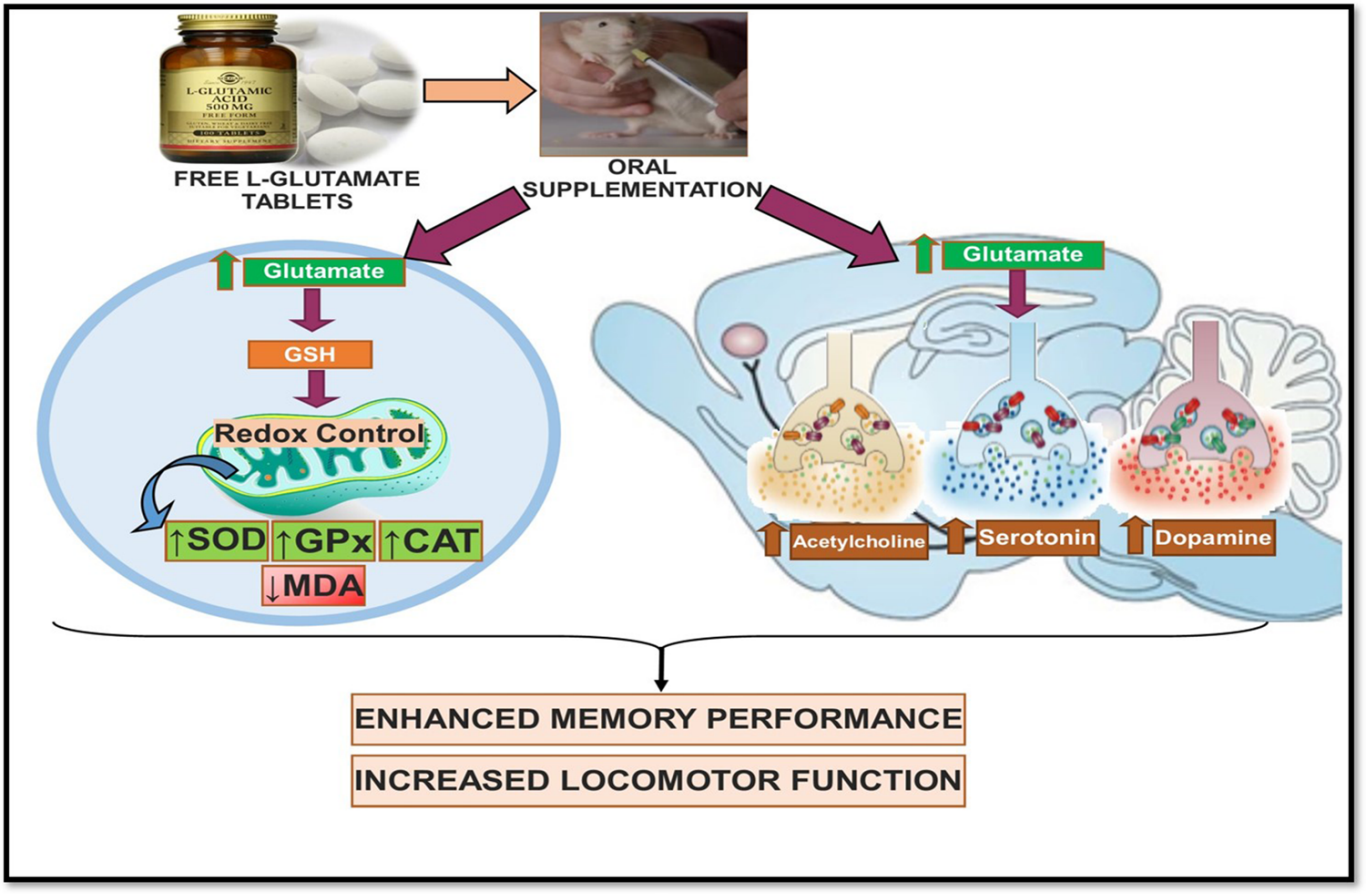
Schematic representation of findings following chronic supplementation of free Glu in rats.

Thus, it can be concluded that chronic supplementation of free Glu effects the behaviour and brain functioning of animals via inducing changes in the oxidative and neurochemical systems in brain (see Fig. 7). In addition to previously reported beneficial effects on periphery, the use of free l-Glu and its beneficial effects in enhancing neurobiological function highlight the novelty of this work. These beneficial effects may be attributed to the improvement in redox homoeostasis and neurotransmission in brain. The findings therefore suggest future supplementation of free l-Glu as a useful therapeutic strategy to combat neurological disorders. However, further targeted studies are needed to elucidate the exact mechanisms behind the efficacy of free l-Glu.

## Materials and methods

### Animals

Locally bred male Albino-Wistar rats (n = 12) utilised in the study, were purchased from Dow University of Health Sciences, OJHA campus, Karachi, Pakistan. Animals housing and handling conditions were same as described previously^60^. Animals were caged individually (to avoid effect of social interaction) with ad libitum access to cubes of standard rodent diet [A control diet (4.47 kcal/g) containing 25% fat, 50% carbohydrate, and 25% protein] and tap water under a 12:12 h light/dark cycle (lights on at 7:00 am) at controlled room temperature (22 ± 2 °C). For seven days prior to the experiment, prior to experiments, animals were subjected to acclimation period and to various handling procedures in order to nullify the psychological affliction of environment for reducing the novelty and handling stress. All animal handling and experimentation were approved by the institutional ethics and animal care committee of the University of Karachi and were conducted under the guidelines of National Institute of Health Guide for the Care and Use of Laboratory Animals^61^. Balanced design was followed during all treatment and behavioural assessment to avoid order and time effect.

### Drugs

Glutamate (Solgar, USA) tablets (available commercially) used during the study were purchased from Kousar Medicos. Analytical grade chemicals used in the study were purchased from Sigma Aldrich, USA and Alfa Aesar, USA. All the reagents for experiments were prepared fresh before starting the experiment. For oral administration drug solutions were made fresh each day. Tablets were dissolved in distilled water. Controls received equal volume of distilled water. Glu was administered at the dose of 103 mg/kg body weight^32^ daily via oral route for 5 weeks in the volume of 0.2 ml/100 g body weight to each rat. The selection of dose was done in accordance with the prescription given on the tablets and from this human recommended dose the animal equivalent dose (AED) was calculated as mentioned by Nair and Jacob,^62^. The period of experimentation was 5 weeks.

### Experimental protocol

Rats (n = 12) (age, 3–4 months, weight, 150–200 g) were divided randomly into two experimental groups each containing 6 rats (n = 6). Control (Group 1) rats received distilled water daily while test (Glu) group received aqueous suspension of glutamate tablets at a dose of 103 mg/kg body weight daily via oral route in a volume of 0.2 ml/150 g body weight to each rat for 5 weeks. At the end of treatment, the behavioural analysis was conducted as presented in the Fig. 1. Behavioural testing paradigms include; Open Field test (OFT) for assessing ambulatory activity, Kondziela’s Inverted screen test (KIST) for assessing muscular strength and Beam walking test (BWT) to determine motor co-ordination, Novel Object Recognition test (NORT) for determining recognition ability, Morris Water Maze test (MWM) and Passive Avoidance task (PAT) for determining spatial reference and associative memory performance. Subsequent to behavioural analysis, rats were decapitated to dissect out their brains from the skull as described by Tabassum et al.^63^. Hippocampus was also dissected out as described previously^60^.

### Behavioral protocols

#### Food intake and body weight

Food intake and body weight of rats was monitored daily during the 5 weeks of the treatment as described previously^32^ (see details in the supplementary file).

### Assessment of locomotor performance

Locomotor performance of rats was assessed by using OFT to assess ambulatory activity, KIST and BWT to determine muscular strength and motor co-ordination. The apparatus and procedures used for all these tasks were exactly similar as described earlier^63^ which is provided in detail in the supplementary file.

### Memory assessment

Memory performance of rats was monitored by using NORT to determine recognition ability, MWM and PAT for assessing the spatial reference and associative memory performance. The apparatus and procedures used for all these tasks were essentially the same as described previously^60^. The details of the procedures are provided in the supplementary file.

### Sample collection

For dissecting out the brain rats were decapitated 24 h after the behavioral analysis and their brains were taken out within 30 s and dipped in ice-cold saline. Thereafter, immediately placed in brain slicer with ventral side up to dissect out the hippocampus as previously mentioned^60,63^ by inserting the blade at into the slots of the brain slicer just above and below the hypothalamus, to cut the brain into three slices which were then shifted to a petri dish placed on ice, moistened with chilled saline (0.9% NaCl). The middle slice was used to dissect out hippocampus bilaterally with the help of sharp scalpel blade. All the brain and hippocampus samples were stored at low temperature (− 20 °C) until biochemical (redox and neurochemical) analysis.

### Biochemical protocols

#### Oxidative status parameters

The tissue homogenate (10%, w/v) was prepared in phosphate buffer (0.1 M, pH 7.4) followed by centrifugation at 12,000 × *g* for 20 min at 4 ◦C for estimating the redox state parameters [lipid peroxidation (LPO) levels, catalase (CAT), glutathione peroxidase (GPx) and superoxide dismutase (SOD) activities, reduced glutathione (GSH) and total protein content] in the same manner as mentioned by Haider et al.^60,64^. For further reading refer to the supplementary file.

### Neurochemical analysis

Frozen brain samples (20%) were homogenized in extraction medium containing 0.4 M PCA (HClO_4_; 70%), sodium meta-bisulfate (0.1%), EDTA (0.1%) and cysteine (0.01%) with the help of an electrical homogenizer using a simple one-step sample preparation method. After homogenization, the samples were placed inside the refrigerator for 15 min to aid the precipitation and then centrifuged at 10,000 rpm for 15 min at 4 °C to precipitate out the protein. The supernatant was collected for determining the GLU, GABA and monoamine content in brain samples. Thereafter, the content of acetylcholine (ACh), Glu and GABA was estimated in the same manner as determined by Tabassum et al.^32^ (For details of all protocols see the supplementary file). The activity of Acetylcholinesterase (AChE) and the concentration of monoamines (NA, DA, 5-HT) and its metabolites (DOPAC, HVA and 5-HIAA) in the brain and hippocampus was determined as described by Haider et al.^60^ (For details of all protocols see the supplementary file). The results were expressed as ng/g of tissue. Along with this, ratios of HVA/DA and 5-HIAA/5-HT are also presented to determine the turnover rate.

### Statistical analysis

The data is presented as mean ± SD and SPSS software version 20.0 was used for the statistical analysis. The data of the behavioural and neurochemical analysis was analysed by Student’s *t*-test. Escape latencies during acquisition trials in MWM were analysed by two-way ANOVA (repeated measures) followed by multiple comparisons by Bonferroni’s test. Results of latency score for Kondziela’s inverted screen test and no of slips during beam walking test was statistically analyzed via Non-parametric (Man-Whitney) analysis. Statistical differences between experimental groups were determined by two-tailed analysis and the significance level was set at *P* ≤ 0.05 for all comparisons.

### Ethical approval

The authors declare that the procedures performed in this study were in accordance with all applicable international, national, and institutional guidelines for the care and use of animals.

## Supplementary information

Supplementary information

## Data Availability

Authors will provide data upon request.
