# Supplementary material for: Free l-glutamate-induced modulation in oxidative and neurochemical profile contributes to enhancement in locomotor and memory performance in male rats
Source: Sci Rep. 2020 Jul 8;10:11206. doi: 10.1038/s41598-020-68041-y (PMC7343824; doi:10.1038/s41598-020-68041-y)
Supplement: Supplementary file 1 — Supplementary information [file 41598_2020_68041_MOESM1_ESM.doc]

# SUPPLEMENTARY INFORMATION:

Free L-Glutamate-Induced Modulation in Oxidative and Neurochemical Profile Contributes to Enhancement in Locomotor and Memory Performance in Male Rats

Saiqa Tabassum^1,2^; Saara Ahmad^3^; Syeda Madiha^1^; Sidrah Shahzad^1^; Zehra Batool^4^; Saida Haider^1^*

# MATERIALS AND METHODS:

## BEHAVIORAL PROTOCOLS:

### Food Intake and Body Weight:

Food intake and body weight of rats was monitored daily during the 5 weeks of the treatment by giving rats a weighed amount of food and weighing the remaining food at next feeding as described by Tabassum et al. (2017). Body weights of the rats were also monitored daily during the five weeks of the treatment. Animals were weighed at the beginning of the experiment and were followed up with daily until the end of this study (Tabassum et al. 2017).

### Assessment of Locomotor Performance:

### *Open field test:*

The open field test is designed to measure behavioural responses such as locomotor activity and exploratory behaviour. The test consists of measuring the activity of rats in an open novel space, from which escape is prevented by a surrounding wall as described by Haider et al. (2016). The apparatus used in this study was consisted of a square area (76 × 76 cm) with opaque walls 42 cm high. The floor is divided into 25 equal squares. Testing was performed in a quiet room under white light. To monitor the activity animals taken out from their home cages were placed in the central square of the open field (one at a time). Activity in open field was determined by monitoring latency period and counting number of squares crossed with all four paws for five minutes as described earlier. Activities of control rats and drug treated rats were monitored alternatively to avoid order effect (Haider et al. 2016).

### *Kondziela's inverted screen test:*

Kondziela's inverted screen test has been used previously for measure of muscular strength using all four limbs (Kondziela, 1964). Nearly all healthy animals easily score maximum on this task. The inverted screen is a 43 cm square of wire mesh consisting of 12 mm squares of 1 mm diameter wire. It is bordered by a 4 cm deep wooden beading (which prevents animals from climbing on to the other side). The test was done by placing the rat in the center of wire mesh screen and the screen was rotated to an inverted position over 120 s with the rat's head declining first. The time when the rat falls off from the screen was noted. Animals were scored for inverted screen as follows: falling between 1 and 10 s = 1, falling between 11 and 25 s =2, falling between 26 and 60 s = 3, falling between 61 and 90 s =4, falling after 90s = 5 (Tabassum et al. 2017).

### *Beam walking test:*

Beam walking is a test of motor coordination (Goldstein and Davis, 1990). The rats have to cross a beam which is suspended between a start platform and their home cage at a height of 50 cm and is supported by two pillars. A cushion was placed under the beam to protect the animals from the bang into the floor. The difficulty of this task can be assorted by using beams with different shapes and widths (Jover et al., 2006). Motor coordination and balance was assessed by the ability of a rat to crossways a graded series of beams. Three circular beams of different diameter were used in this study such as 3 cm, 2 cm, 1 cm and length of 100 cm. In the training phase animals were trained to traverse the beam (from widest to narrowest) directly into the animal's home cage. This helps to make certain that the behavior during testing is more stable and more precisely reflects motor coordination as opposed to the rodent's natural aversion to crossing over unprotected spaces. After training session testing phase was done, the time taken to cross the beam and number of foot slips off the beam was determined. The foot slips off the beam made by rat was scored on a scale of 0–6 as follows: 0 points, the rat was not able to stay on the beam, 1 point; the rat did not move but able to stay on beam, 2 points; the rat tried to traverse beam but fell, 3points; the rat traversed the beam with multiple slips (4–6), 4 points; the rat traversed the beam with few foot slips (2 or 3), 5 points; the rat traversed the beam with only one slip of the hind limb, 6 points; the rat traversed the beam without any slip of hind limb (Puurunen, 2001; Tabassum et al. 2017).

### Memory Assessment:

### *Object recognition task:*

The novel object recognition test was used to assess the cognitive ability of animals. It was established by Ennaceur and Delacour in 1988 to assess the rat’s ability to distinguish a novel (new)object in a familiar environment (Ennaceur and Delacour, 1988). This is a test of recognition memory. The procedure performed in this experiment was same as described by Okuda which comprised of exposing rats to two similar objects and then recording their discriminating ability to discriminate the novel object placed at the same time along with one of familiar objects (Okuda et al.,2004). In this test the method used was essentially the same as that Okuda et al. (2004) with slight modifications as explained by Haider et al. (2016). The apparatus consists of square box made of grey painted wood having dimensions of 45 × 45 × 45 cm3. In order to saturate it with olfactory stimuli, cleaning of box was not allowed throughout the experiment. The objects to be discriminated were two similar transparent glasses filled with white cement (used as familiar objects, A1 and A2) in order to make them heavy enough so that rats could not be able to move them, and a metallic container of same size filled with white cement (used as a novel object, B). The size of the objects was 2.5times the size of the rat so that the rat could easily sniff it. The rat was placed in the middle of the box facing the wall and was allowed to explore the apparatus and the objects. The test was per-formed in three phases; habituation, training, and test session. On day one in order to habituate the rats were pre-exposed to the testing chamber for 10 min. Next day 24 h after pre-exposure rat was positioned inside the box with two similar objects (A1 and A2) and was allowed to explore the objects for 5 min. After 5 min, rat was removed from the object recognition box and returned to its home cage. Objects A1 and A2 were also removed from the box. Twenty minutes were allowed to elapse before the start of the 3 min test phase (Cohen et al., 2013). During the test phase, rat was removed from home cage and returned to the object recognition box, where it was exposed to one new object (B) and one of the old objects for 3 min. The sniffing time for the novel and familiar object was measured. Exploration of an object was defined as when the rat directed its nose toward the object at a distance of <2 cm. Reports have shown that no difference in exploration for two objects at the test phase can be interpreted as a memory deficit (Haider et al.,2007; 2016). The total exploration time of the familiar and novel objects were used to calculate discrimination index to evaluate cognitive performance as described by Okuda et al. (2004). The discrimination index is an index of measure of discrimination between the familiar and the novel objects corrected for exploratory activity. Itis calculated as: (time spent on novel object−time spent on familiar object)/(time spent on novel object + time spent on familiar object).The discrimination index can range from −1 to 1, with −1 indicating complete preference for the familiar object, 0 indicating no preference for either object, and 1 indicating complete preference for the novel object (Haider et al. 2016).

### *Morris water maze test (MWM):*

The Morris water maze (MWM) is a behavioral test that is frequently employed to test numerous aspects of learning and memory in rodents. MWM test was performed to examine the effects on spatial memory. It is used to assess hippocampal-dependent spatial learning and memory. It was developed by Richard G. Morris in 1981 (Morris, 1981). This test requires an ani-mal to use spatial learning and memory to locate a hidden platform just below the surface of a circular pool of water and also to remember its location as in the previous trial. The maze used for rats is same as described by Srikumar et al. (2004) with some modifications as explained by Haider et al. (2016). It is a circular pool of water with a diameter of 45 cm, height37 cm and depth of water is 12 cm. The pool is a metal cylinder painted white on the inner surface and the escape platform is also made of metal cylinder with flat metallic top having a surface diameter of 8 cm and is 2 cm below the surface of water during water maze training. The pool is filled with water (23 ± 2◦C) and made opaque with milk in order to obscure the platform and to allow proficient tracking of the swim paths of the rats. Animals placed in the circular pool have to swim until they find a hidden platform submerged in the pool. Learning and memory are measured by escape latency and time spent in the target quadrant in which the platform is hidden. It is reported that the animal uses cues in order to locate the hidden platform. The performance of animals improved across trials (indicated by a reduction in latencies to locate the platform) despite starting from multiple locations. Such a procedure mitigates the use of egocentric navigation and promotes the use of allocentric navigation. Each trial started by placing the rat gently in the water, facing the edge of the circular pool, at the designated position. After learning training, rat was given a probe trial in which the platform was removed and memory for platform location was assessed by quantifying the time spent in the maze quadrant that previously contained the platform (‘target quadrant’). The Morris water maze task comprised of three components: In order to familiarize the rats with the maze and the escape procedure the training session was performed initially during which each rat was placed into the water in such a way that their face was towards the wall of the tank. After placing, 120 s were given to each animal to find and mount onto the hidden platform, if the rat located the platform it was allowed to stay on it for 10 s. If it failed to locate the platform during the allocated time, then it is usually picked up and was guided gently onto the platform (Haider et al.,2011; 2016) and placed on it for ∼15 s. Place or spatial learning is the most basic MWM procedure. The concept behind it is that the animal must learn to use distal cues to navigate a direct path to the hidden platform when started from different, random locations around the perimeter of the tank. If there are no proximal cues available, the use of distal cues provides the most effective strategy to accomplish this. Each rat was given four consecutive trials in the maze to locate the hidden platform with the hidden escape platform (10 cm diameter) being located in the NW quadrant, 2 cm below the water surface. The interval between trials was 15 min. Starting positions from arbitrarily assigned compass locations were randomized foreach rat on each trial. We used four start locations: NE, SE, NW and SW (target quadrant). These positions are designed so that the animal is not able to learn a specific order of right or left turns to locate the platform, while using each of the four start positions. The platform was kept in a constant position for all rats throughout all trials. The time taken to reach the platform (escape latency) was noted. Probe Trial: To assess reference memory at the end of learning, a probe (transfer) trial was performed. In the present study we have performed two probe trials, one was performed 1 h after the last acquisition trial and the other was performed after 24 h of last hidden platform acquisition trial. During the probe trial, the platform was removed, and the rats were allowed to swim for 1 min. The parameters that were measured during this trial included the time spent in the target quadrant (NW) and the number of entries over the target quadrant (NW) as an index of reference memory (Haider et al., 2016).

### Passive avoidance test (PA):

The Passive Avoidance task is a fear-aggravated test used to evaluate learning and memory in rodents. Passive avoidance is an operant conditioning task where an animal learns to suppress movement in order to avoid an aversive stimulus. Passive avoidance test is used to assess memory function based on the association formed between an aversive stimulus such as a mild foot shock and a specific environmental context. In this test, rat learn to avoid an environment in which an aversive stimulus (foot-shock) was previously delivered. The rat can freely explore the light and dark compartments of the chamber and a mild foot shock is delivered in one side of the compartment. Rat eventually learn to associate certain properties of the chamber with the foot shock. The latency to pass the gate in order to avoid the stimulus is used as an indicator of learning and memory (Khaliq et al., 2008; Haider et al., 2016). Passive avoidance paradigm consists of two compartments as an illuminated ‘safe’ and a dark ‘punishable’ one. Both compartments were connected with a door that enable free crossing from one compartment to another. Both compartments have a grid floor. The diameter of rods is 5 mm with 0.5 cm distance between the rods. The Passive avoidance task was comprised of two sessions; training and testing. In the training session, rat was placed in an illuminated box. Once the rat prompted by its instinct stepped with its four paws into the dark compartment, the door was closed, and rat received 1.5 mA foot-shock through the grid floor to its paws for 5 s. After receiving the foot shock, it was allowed to stay in this compartment for 10 s after that the door was opened and it immediately returned back to illuminated safe compartment. During the training session the initial step-through latency to enter the dark compartment was noted with the help of stopwatch. After a variable delay (minutes to days), the animal undergoes a test session. During the test period, rat was placed in the bright compartment again after training session with cutoff time of 3 min. The step- through latency that indicates the time elapsed before the rat entered the dark compartment was recorded in the test session with the help of stopwatch. The test session was further divided into two phases; acquisition and retention phase. In the acquisition phase, the rat was returned to the brightly lit compartment again after 60 min (1 h) of last learning trial, and the latency to enter the shock-paired compartment was measured (recall latency). While in the retention Phase, the rat was returned to the brightly lit compartment after 24 h (1 day) of last learning trial, and the latency to enter the shock-paired compartment was measured (retention or recall latency) (Haider et al. 2016). The rat that learned the task would avoid the location previously paired with the aversive stimulus and show greater latency to enter it. The decline in step-through latencies to enter the dark compartment in the test session were treated as a measure of “amnestic” effects. The decline in the difference of post-training to pre-training step-down latency was computed for each animal and served as an index of memory deficit (Wass et al., 2013). Avoidance memory/associative memory performance of rats was assessed by recording the step-through latencies during initial training trial, during acquisition phase performed after 60 minutes of initial training trial and during retention phase performed after 24hr of initial training trial and then the difference of step-trough latency was computed for each animal as an index of memory deficit (Haider et al. 2016).

## OXIDATIVE STATUS PARAMETERS:

***Determination of oxidative agent (LPO):***

Estimation of LPO was performed as described by Chow and Tappel (1972) with slight modifications as mentioned by Haider et al. (2014, 2015; 2016) via determining the levels of Malondialdehyde (MDA). Brain homogenate (300 μl) was added into a TCA (15%)–TBA (0.375%) mixture (2 ml). The mixture was boiled for 20 min in a water bath and cooled immediately which is then centrifuged at 2000g for 10 min. A supernatant of light pink color was then collected, and the absorbance was recorded at 532 nm. Lipid peroxidation was quantified using molar extinction coefficient (1.56×10^5^) and data is represented as nmoles of MDA/g of brain tissue (Haider and Tabassum, 2018).

***Determination of activity of superoxide dismutase (SOD):***

Determination of SOD activity in brain was based according to the method of Beauchamp and Fridovich (1971) as described by Chidambara Murthy, Jayaprakasha, and Singh (2002) and Haider et al. (2014, 2015, 2016). The method determines the reduction ability of NBT to form a water-insoluble compound, blue formazan. Brain homogenate (0.5 ml) was mixed with Na2CO3 (1 ml of 50 mM), NBT (0.4 ml of 24 μM), and EDTA (0.2 ml of 0.1 mM). After mixing hydroxylamine hydrochloride (0.4 ml of 1 mM) was added to start the reaction. The absorbance was recorded immediately at 0 min till 10 min with consecutive intervals of 1 min at 560 nm at 25 °C. The change in absorbance was recorded at time zero (0 min) and after 10 min. An appropriate control without brain homogenate was run along each batch of samples and used to determine the percent inhibition of NBT reduction. The specific activity of SOD was presented as U/g of brain tissue. 1 unit is equal to the quantity of the enzyme that inhibits NBT reduction by 50% (Haider and Tabassum, 2018).

***Estimation of catalase (CAT) activity:***

Activity of CAT was determined as described by Sinha (1972) and Haider et al. (2014, 2015, 2016). Brain homogenate (0.1 ml) was added into the reaction mixture containing 0.01M phosphate buffer at pH 7.4 (1 ml) and 0.2M H2O2 (0.4 ml). After that, the mixture in tubes was incubated at 37 °C for 15 min in a water bath. After incubation, a potassium dichromate-acetic acid reagent (2 ml of 5%) was added to stop the reaction and then mixture was read immediately at 570 nm against reagent blank to determine how much H2O2 was consumed. An appropriate control was carried out containing H2O2 without the addition of brain homogenate. CAT activity was quantified using molar extinction coefficient (71) and presented in terms of consumption of H2O2 as μmol/min/g of brain tissue (Haider and Tabassum, 2018).

***Estimation of glutathione peroxidase (GPx) activity:***

GPx activity was assessed by the method of Flohe and Gunzler (1984) as described by Haider et al. (2014, 2015). The reaction mixture containing phosphate buffer (0.1 M, pH 7.4), reduced glutathione (2 mM, 0.2 ml), sodium azide (10 mM, 0.1 ml) and H2O2 (1 mM, 0.1 ml) was mixed with brain homogenate (0.3 ml). Then the mixture was incubated for 15 min at 37 °C in a water bath. After incubation, TCA solution (0.5 mil, 5%) was added into the mixture to terminate the reaction. The mixture was then centrifuged for 5 min at 1500g for 5 min and the supernatant was collected. After that, 0.1 of supernatant was mixed with 0.1M phosphate buffer (0.2 ml, pH 7.4) and 5, 5-dithiobis (2-nitrobenzoic acid) (DTNB) (0.7 ml, 0.4 mg/ml) and then absorbance was measured at 420 nm against the reagent blank prepared without addition of reduced glutathione. For estimating the enzyme activity an appropriate control was also carried out containing reduced glutathione without addition of brain homogenate. Activity of GPx was quantified using molar extinction coefficient (6.22×10^3^) and was presented as μmol/min/g of brain tissue (Haider and Tabassum, 2018).

**Estimation of reduced glutathione (GSH):**

Brain GSH content was determined by its reaction with 5, 5-dithiobis (2-nitrobenzoic acid) (DTNB) (Ellman’s reagent) to yield a yellow chromophore which was measured spectrophotometrically as described by Ellman (1959) and Haider et al. (2016). The brain homogenate (0.3 ml) was mixed with an equal amount of 15% Perchloric acid (HClO4; PCA) and stored overnight at 4 °C (Stempak et al., 2001). PCA treated samples were then centrifuged at 5000 rpm for 10 min at 4 °C. The supernatant was collected for GSH estimation while pallets were used for protein assay. The 0.1 ml of supernatant was mixed with reaction mixture containing 0.1M phosphate buffer (2 ml, pH 8.4), DTNB-NaHCO3 mixture (0.5 ml) and 0.4 ml of double-distilled water. The mixture was shaken vigorously on vortex and the absorbance was read at 412 nm within 15 min against reagent blank containing reaction mixture. The GSH content was determined using molar extinction coefficient (13600) and data is represented as nmol/g of brain tissue (Haider and Tabassum, 2018).

***Determination of protein content in brain***

The protein content in brain samples was estimated using a previously reported method of Lowry, Rosebrough, Farr, and Randall (1951) as described by Haider et al. (2016). The pallet obtained during GSH estimation following PCA precipitation was used for determination of protein content. The precipitate (pallet) was dissolved in 1 ml of NaOH (0.1 N) by vigorous mixing. After that mixture was set aside for 10 min at room temperature, then 0.5 ml of Follin reagent (Sodium 1,2- napthoquinonoe-4-sulfate) was added into the mixture. The mixture was then again incubated for 10 min at room temperature for complete colour development. The absorbance was measured at 610 nm against reagent blank. The concentration of protein was calculated using standard bovine serum albumin (BSA) solution (200 mg/dl) and data is represented as g of protein/g of brain tissue (Haider and Tabassum, 2018).

## NEUROCHEMICAL ANALYSIS:

***Determination of brain acetylcholine (ACh) content:***

ACh content in brain samples was estimated according to the method of Hestrin (1949) with modifications as described by Tabassum et al. (2017). The brain tissue (0.08 g) was boiled in pre-heated tubes in a boiling water bath for 10 min to inactivate the enzyme so that bound ACh was released. The boiled tissue was then homogenized in distilled water (0.8 ml) using an electrical homogenizer and then 1 ml of TCA (1.84 M) was added into the homogenate. The 0.4 ml of homogenate was then mixed with an equal volume of alkaline hydroxylamine hydrochloride (14%) solution and incubated at room temperature for 10 min. After incubation, dilute HCl (0.4 ml, 50%) was added into the homogenate mixture and centrifuged at 3500 rpm for 10 min at 4 °C. The supernatant was collected and mixed with 0.2 ml of acidic ferric chloride solution (0.37 M) resulting in reaction of ACh with ferric chloride to develop brown color that read immediately at 540 nm against the reagent blank. The tissue ACh content was determined using molar extinction coefficient (1005) and is represented as nmol/g of tissue (Haider and Tabassum, 2018).

***Determination of acetylcholinesterase (AChE) activity:***

Acetylcholinesterase (AChE) activity in the brain was estimated as described by Ellman, Courtney, Andres, and Featherstone (1961) and Haider et al. (2014) using acetylthiocholine (ATC) as a substrate. Brain samples were homogenized (2%) in 0.1M phosphate buffer (pH 8.0) using an electrical homogenizer. Brain homogenate (0.4 ml) was then added to a reaction mixture containing 0.1M phosphate buffer (2.6 ml, pH 8.0), and DTNB (0.1 ml) and mixed by bubbling air placing inside the spectrophotometer. Once the reaction became stable, the absorbance was measured at 412 nm for the basal reading against the air blank. After that to start the enzyme reaction ATC (5.2 μl) was added and the absorbance was recorded again for 10 min at the intervals of 1 min at 25 °C. The average difference in absorbance was computed between time zero and 10 min (Haider and Tabassum, 2018). The activity of AChE was quantified using molar extinction coefficient (1.36×10^4^) and was presented as nmol/min/g of brain tissue (Haider and Tabassum, 2018).

***Extraction procedure for determination of amino acids and monoamines:***

Frozen brain samples (20%) were homogenized in extraction medium containing 0.4M PCA (HClO4; 70%), sodium meta-bisulfate (0.1%), EDTA (0.1%) and cysteine (0.01%) with the help of an electrical homogenizer using a simple one-step sample preparation method (Haider and Tabassum, 2018). After homogenization, the samples were placed inside the refrigerator for 15 min to aid the precipitation and then centrifuged at 10,000 rpm for 15 min at 4 °C to precipitate out the protein (Haider and Tabassum, 2018). The supernatant was collected for determining the GLU, GABA and monoamine content in brain samples.

***Estimation of glutamate (GLU) content:***

GLU content in brain is estimated using commercially available Cell Biolabs’ Glutamate Assay Kit (STA-674) which is based on a quantitative fluorometric assay in the same manner as described by Tabassum et al. (2017). The kit has a detection sensitivity limit of 300 nM glutamate. The assay uses glutamate-specific enzymes (i.e. Glutamate oxidase (0.08 U/mL) that converts glutamate to α-ketoglutarate and produces NH_3_ as well as H_2_O_2_, and glutamate-pyruvate transaminase (0.5 U/mL) with L-alanine (200 μM) that regenerates glutamate providing significant amplification of H2O2 production that reacts with sample (50 μl) to generate H2O2. In the presence of horseradish peroxidase (HRP) (0.2 U/mL), the Fluorometric Probe (100 μM) reacts with H2O2 to produce highly fluorescent Resorufin which is incubated for 30–45 min at 37 °C in dark. The Resorufin product can be easily read by a fluorescence microplate reader with an excitation of 530–560 nm and an emission of 590 nm. Fluorescence values are proportional to the GLU levels within the samples. The concentration of GLU within samples is determined by comparing the sample RFU with a standard curve obtained by using L-Glutamate Standard (20mM solution) and expressed as mmol/g of tissue (Haider and Tabassum, 2018).

***Estimation of GABA content:***

Concentration of GABA in brain is estimated using commercially available Gamma-aminobutyric acid ELISA kit (Bioassay Technology Laboratory, China) in the same manner as described by Tabassum et al. (2017). The detection range of this kit is 2μg/dl - 600μg/dl of GABA. The During the assay, GABA (sample, 40 μl) is added to the wells pre-coated with GABA monoclonal antibody. Then a biotin-conjugated anti-GABA antibody (10 μl) is added that binds to GABA. After that Streptavidin-HRP (50 μl) is added and binds to the biotin-conjugated anti-GABA antibody. The plate was then incubated for 60 min at 37 °C. After incubation, a washing step was done to wash away unbound Streptavidin-HRP. After washing substrate solutions were added and again incubated for 10 min at 37 °C in the dark for developing color proportional to the GABA content. The acidic stop solution was then added to terminate the reaction and absorbance is measured at 450 nm using a microplate reader. Concentration of GABA within samples is determined by comparing the sample O.D with a standard curve obtained by using GABA Standard (640 μg/dl) solution and expressed as μmol/g of tissue (Haider and Tabassum, 2018).

***Determination of monoamines:***

Concentrations of monoamines and their major metabolites [norepinephrine (NA), 3,4-dihydroxyphenyl acetic acid (DOPAC), dopamine (DA), homovanillic acid (HVA), 5-hydroxyindoleacetic acid (5-HIAA), and 5hydroxytryptamine (5-HT)] in brain samples was estimated by using reversed phase high performance liquid chromatography with electrochemical detector (HPLC-EC) in the same manner as described by Haider et al. (2016). A 5 μ Shim-Pack C18 ODS (Octadecyl silane) separation column of 4.0mm internal diameter and 150mm length was used as a stationary phase. Separation was achieved by a mobile phase containing methanol (14%), anionic detergent Octyl sodium sulphate (OSS) (0.023%) and ethylenediaminetetraacetic acid (EDTA) (0.0035%) in 0.1M phosphate buffer of pH 2.9 at an operating pressure of 2000–3000 psi on Schimadzu HPLC pump at the flow rate of 1 ml/min at 35 °C. Electrochemical detection was achieved on Schimadzu LEC 6A detector with glossy carbon electrode at an operating potential of +0.8 V vs Ag/AgCl as a reference electrode (Haider and Tabassum, 2018). This method can detect monoamines in the range of 10ng/ml to 600ng/ml. The data obtained by HPLC-EC system was recorded and quantified using LC-solutions software version 2005. Concentrations of NA, DOPAC, DA, HVA, 5-HIAA and 5-HT was computed using an external standard solution containing NA (100 ng/ml), DA (100 ng/ml), DOPAC (100 ng/ml), HVA (100 ng/ml), 5-HIAA (100 ng/ml) and 5-HT (200 ng/ml) and expressed as ng/g of brain.
